# Simultaneous Cesarean Section and Maternal Cardiac Surgery: Outcomes and Feasibility from a Tertiary Care Hospital in India

**DOI:** 10.21470/1678-9741-2022-0335

**Published:** 2023-07-18

**Authors:** Pooja Sikka, Vidur Bansal, Sachin Mahajan, Pankaj Aggarwal, Sanjeev Hanumantacharya Naganur

**Affiliations:** 1 Department of Obstetrics, Postgraduate Institute of Medical Education and Research, Chandigarh, India; 2 Department of Cardiothoracic and Vascular Surgery, Postgraduate Institute of Medical Education and Research, Chandigarh, India; 3 Department of Cardiology, Postgraduate Institute of Medical Education and Research, Chandigarh, India

**Keywords:** Pregnancy, Cesarean Section, Maternal Mortality, Cardiovascular Diseases, Peripartum Period, Sitting Position, Heart Failure

## Abstract

**Introduction:**

Cardiovascular disease is the leading cause of pregnancy-related mortality, and it has gradually increased over time; this rise has been attributed to numerous reasons including the growing number of women with congenital heart disease who are surviving to childbearing age. Valve surgery during pregnancy is a high risk, with a fetal and maternal mortality rate of 35% and 9%, respectively. Prior knowledge about the cardiovascular disease opens up a host of options for the mother even during pregnancy, but presentation in the 3rd trimester puts both the mother and the baby at risk. Simultaneous caesarean section and maternal cardiac surgery is a suitable option for this subset of patients, and with this study we aim to assess its outcomes and feasibility.

**Methods:**

This is a retrospective study of five pregnant patients who presented with predominant symptoms of heart failure in the 3^rd^ trimester between June 2019 and June 2021. Intraoperative and postoperative intensive care unit charts of all the patients were reviewed.

**Results:**

All five patients underwent simultaneous cesarean section and maternal cardiac surgery successfully with no fetal or maternal mortality and are doing well in the follow-up period.

**Conclusion:**

Cesarean section followed by definitive maternal cardiac surgery in the same sitting is a safe and feasible approach in the management of such patients. A well-prepared team is pivotal for a safe delivery with a cardiopulmonary bypass machine on standby. Specialized multidisciplinary care in the antepartum, peripartum, and postpartum period is essential to improve outcomes.

## INTRODUCTION

Cardiovascular disease is the leading cause of pregnancy-related mortality, and it has gradually increased over time (from 7.2 to 17.2 deaths per 1,000,000 live births from 1987 to 2015). The rise in maternal mortality has been attributed to numerous reasons including the growing number of women with congenital heart disease who are surviving to childbearing age^[1]^. Valve surgery during pregnancy is a high risk, with a fetal and maternal mortality rate of 35% and 9%, respectively^[2]^.

Congenital heart disease accounts for most antenatal heart disease cases in high-income, industrialized countries^[3]^. This pattern differs in low- and middle-income countries, where 88%–90% of antenatal heart diseases are attributable to rheumatic heart disease (RHD). RHD is the most common cardiac disease during pregnancy in India with mitral valve being most commonly affected, and it is an important cause of maternal mortality in our country^[4]^. Optimal management of patients who present late in the 3rd trimester with symptoms of heart failure is a challenge with sparse literature throwing light on it. Early and specialized multidisciplinary care in the antepartum, peripartum, and postpartum period is essential to improve cardiovascular outcomes and to reduce maternal mortality, while, at the same time, safeguarding the health of the baby. Here, we report the outcomes of five patients who underwent successful caesarean section and maternal cardiac surgery in the same sitting.

## METHODS

Five patients without any prior knowledge of their heart disease presented to the emergency department with symptoms of heart failure in the 3rd trimester of pregnancy. All patients had their irregular antenatal visits during the 1st and 2nd trimesters, but none of them had been diagnosed to have a cardiac disease, possibly because of lack of facilities at a rural environment. All patients underwent simultaneous cesarean section and cardiac surgery between July 2019 and July 2021 at a tertiary care centre in North India after a detailed discussion by the Heart Team, including an obstetrician.

After extensive discussion by the Heart Team, a common consensus was reached regarding the management in every single case. In all these five patients, a lower segment cesarean section (LSCS) was done first to safeguard the life of the baby. A cardiac surgery team was with the cardiopulmonary bypass (CPB) pump on standby to intervene in case a mishap happened during the LSCS. Following an uneventful LSCS in all the patients, attention was turned towards addressing the cardiac pathology. Postpartum hemorrhage in such patients would not have been tolerated, so all patients were started on oxytocin infusion without any boluses. Owing to the cardiovascular side-effects, other uterotonic agents were not used. Oxytocin infusion was prepared by diluting 20 units in 500 ml normal saline and was started at 125 ml/hour. This infusion was continued for 24 hours in all patients.

After a median sternotomy and standard bicaval and aortic cannulation, the heart was arrested in diastole using antegrade blood cardioplegia. The pathology was corrected as described below. The patients were subsequently weaned off CPB and shifted to the intensive care unit (ICU) on routine ionotropic support. None of the patients had any complications in the postoperative period. All the patients are doing well in the follow-up period. All the neonates could be discharged home at an average of eight days following LSCS.

## RESULTS

All five patients presented during their 3^rd^ trimester without any prior knowledge of the heart disease they were suffering from (Table 1). Although two patients had a prior successful pregnancy, they were unaware of the cardiac pathology. The main symptom of all the patients was refractory heart failure, that was not responding to medical management. All decisions were taken in a Heart Team meeting. Both the fetal and maternal health were important, but the maternal health condition was prioritized. As a result, all the patients were planned for a cesarean section followed by the maternal cardiac surgery (Table 2). In two patients, this process had to be rushed and the cesarean and maternal cardiac surgery were performed in an emergency setting at 31 and 34 weeks, respectively. In the rest of the three patients, a semi-elective surgery was performed after consultation with the neonatologist, keeping in mind the fetal maturity. Mean age of presentation was 26.6 years and the mean period of gestation at the time of surgery was 35 weeks. Three patients had a bicuspid aortic valve and severe aortic stenosis; these patients underwent an aortic valve replacement with a mechanical bileaflet prosthesis. One patient had a ruptured sinus of Valsalva (RSOV) involving the right coronary cusp with a subpulmonic ventricular septal defect (VSD), and she underwent a successful RSOV repair along with closure of the subpulmonic VSD with a polytetrafluoroethylene patch. One patient had large patent ductus arteriosus (PDA) along with severe aortic regurgitation. She underwent PDA ligation and aortic valve replacement with a mechanical bileaflet prosthesis under deep hypothermic circulatory arrest. The mean ICU stay was 4.6 days, and mean ward stay was 5.4 days. The mean cross-clamping time was 61.4 minutes with a mean CPB time of 97.8 minutes. All patients along with their babies were successfully discharged home and are currently doing well in the follow-up period.

**Table 1:** Patient characteristics.

Subject number	Age (years)	Period of gestation at time of surgery (weeks)	Gravida	Diagnosis	Surgery	Postoperative ICU stay (days)	Postoperative ward stay (days)
1	30	38	Multigravida	Severe aortic stenosis	Aortic valve replacement (#19 mm, ATS, mechanical bileaflet valve)	3	5
2	26	36	Multigravida	Severe aortic stenosis	Aortic valve replacement (#19 mm, ATS, mechanical bileaflet valve)	4	7
3	22	31	Primigravida	RSOV with subpulmonic VSD	RSOV repair with VSD closure	7	6
4	25	34	Primigravida	Severe aortic stenosis	Aortic valve replacement (#21 mm, SJM, mechanical bileaflet valve)	5	5
5	30	36	Primigravida	Large PDA with severe aortic regurgitation	PDA ligation with aortic valve replacement (#21 mm, SJM, mechanical bileaflet valve)	4	4

ATS=Advancing the Standard; ICU=intensive care unit; PDA=patent ductus arteriosus; RSOV=ruptured sinus of Valsalva; SJM=St. Jude Medical; VSD=ventricular septal defect

**Table 2:** Intraoperative details.

Patient	Prior cesarean section	Cross-clamping time (minutes)	CPB time (minutes)	Minimum temperature during surgery (°C)
1	Semi-elective	45	68	32
2	Semi-elective	50	76	32
3	Emergency	78	125	32
4	Emergency	54	80	32
5	Semi-elective	80	140	24

CPB=cardiopulmonary bypass

## DISCUSSION

Valvular heart disease pathologies in women of childbearing age are most commonly congenital but may include rheumatic, acquired, and native degenerative causes. The recently published American College of Gynaecology (ACOG) guidelines recommend the estimation of risk and subsequent management with the modified World Health Organization (or WHO) classification^[5]^. CPB during pregnancy poses maternal and fetal risk, and although maternal mortality rate has been found to be the same as in non-pregnant woman in the current era, fetal mortality is still high^[6]^.

CPB in the immediate postpartum period might result in severe uterine bleeding due to high dose of heparin and use of anesthetic drugs which cause uterine relaxation. Keeping this in mind, we undertook all the precautions and none of the cases had significant bleeding. Continuous infusion of oxytocin and per rectal misoprostol helped in maintaining uterine tone and minimized bleeding during the cardiac procedure. Further care was taken to achieve meticulous hemostasis before closing the abdomen by re-inspection of uterine incision and tone. Ideally, severe valvular heart disease should be treated before conception and consideration should be given to performing valve repair or replacement with a bioprosthetic valve to minimize the risks associated with therapeutic anticoagulation during future pregnancies albeit at the increased risk of structural valve degeneration^[7]^.

In our series, we chose mechanical prosthesis for valve replacement. This was decided after a detailed discussion in the Heart Team meeting along with the patient and her family. Our preference was more towards a mechanical prosthesis because of the increased risk of structural valve degeneration with the bioprosthetic valve. Also, most of the patients’ family members were apprehensive of another surgery, which could pose a major financial and emotional burden.

Some congenital diseases may go unrecognised till adulthood when they are incidentally diagnosed during routine evaluation. All five of our patients had a congenital disease that was not recognised in early childhood and was diagnosed for the first time during pregnancy. Three out of the five patients had a bicuspid aortic valve, one patient had PDA, and one patient had a subpulmonic VSD.

Cesarean delivery can be achieved in the shortest time possible and provides a more controlled environment for monitoring the mother’s condition with complex physiological changes taking place in her body during labour. Cardiovascular disease complicates this physiology further and intensive monitoring is required to prevent maternal collapse, thus favouring cesarean delivery over normal vaginal delivery.

The ACOG recommends elective induction of labour for pregnant women with cardiac disease between 39 and 40 weeks of gestation in patients who do not have spontaneous onset of labour or clinical indications for preterm delivery. The timing of delivery for women with active, maternal, or fetal conditions is highly variable according to the underlying medical problem^[4]^. Preterm birth and low-birth-weight babies are known as the major neonatal complications in women with heart disease during pregnancy. In our series, four out of the five babies were small for gestational age, and one baby was delivered pre-term.

## CONCLUSION

Management of pregnant patients with cardiovascular disease is a challenge with limited experience. Cesarean section followed by definitive maternal cardiac surgery in the same sitting is a safe and feasible approach in the management of such patients. A well-prepared team is pivotal for a safe delivery with a CPB machine on standby.

Specialized multidisciplinary care in the antepartum, peripartum, and postpartum period is essential to improve outcomes. Only a handful of case reports have discussed the management of such patients in case of an emergency in the terminal part of the pregnancy. This case series, being the largest of its kind, aims to highlight the management in such emergencies.

## Abbreviations, Acronyms & Symbols

ACOG: American College of Gynaecology
ATS: Advancing the Standard
CPB: Cardiopulmonary bypass
ICU: Intensive care unit
LSCS: Lower segment cesarean section
PDA: Patent ductus arteriosus
RHD: Rheumatic heart disease
RSOV: Ruptured sinus of Valsalva
SJM: St. Jude Medical
VSD: Ventricular septal defect
